# Male engagement guidelines in antenatal care: unintended consequences for pregnant women in Tanzania

**DOI:** 10.1186/s12884-021-04141-5

**Published:** 2021-10-26

**Authors:** Haika Osaki, Saumya S. Sao, Godfrey A. Kisigo, Jessica N. Coleman, Rimel N. Mwamba, Jenny Renju, Blandina T. Mmbaga, Melissa H. Watt

**Affiliations:** 1 Kilimanjaro Clinical Research Institute, Moshi, Tanzania; 2 Duke Global Health Institute, Duke University, Durham, NC USA; 3 Department of Psychology and Neuroscience, Duke University, Durham, NC USA; 4 Department of Epidemiology and Biostatistics, Kilimanjaro Christian Medical University College, Moshi, Tanzania; 5 Department of Population Health, London School of Hygiene and Tropical Medicine, London, UK; 6 Department of Population Health Sciences, University of Utah, Salt Lake City, UT USA

**Keywords:** Antenatal care, Prevention of mother-to-child transmission, HIV, Male engagement, Maternal health, Partner support, United Republic of Tanzania

## Abstract

**Background:**

The meaningful engagement of male partners in antenatal care (ANC) can positively impact maternal and newborn health outcomes. The Tanzania National Plan for the Elimination of Mother to Child Transmission of HIV recommends male partners attend the first ANC appointment as a strategy for HIV prevention and treatment. This recommendation seeks to increase uptake of HIV and reproductive healthcare services, but unintended consequences of these guidelines may negatively impact women’s ANC experiences. This study qualitatively examined the impact of policy promoting male engagement on women’s ANC experiences.

**Methods:**

The study was conducted in two urban clinics in Kilimanjaro Region, Tanzania. In-depth interviews were conducted with 19 participants (13 women and 6 male partners) attending a first ANC appointment. A semi-structured guide was developed, applying Kabeer’s Social Relations Approach. Data were analyzed using applied thematic analysis, combining memo writing, coding, synthesis, and comparison of themes.

**Results:**

Male attendance impacted the timing of women’s presentation to ANC and experience during the first ANC visit. Women whose partners could not attend delayed their presentation to first ANC due to fears of being interrogated or denied care because of their partner absence. Women presenting with partners were given preferential treatment by clinic staff, and women without partners felt discriminated against. Women perceived that the clinic prioritized men’s HIV testing over involvement in pregnancy care.

**Conclusions:**

Study findings indicate the need to better assess and understand the unintended impact of policies promoting male partner attendance at ANC. Although male engagement can benefit the health outcomes of mothers and newborn children, our findings demonstrate the need for improved methods of engaging men in ANC. ANC clinics should identify ways to make clinic settings more male friendly, utilize male attendance as an opportunity to educate and engage men in pregnancy and newborn care. At the same time, clinic policies should be cognizant to not discriminate against women presenting without a partner.

**Supplementary Information:**

The online version contains supplementary material available at 10.1186/s12884-021-04141-5.

## Introduction

Male engagement in reproductive and maternal health care plays an important role in improving health outcomes among pregnant women and newborn children [1]. Globally, a man’s role in the household often centers around decision making and the provision of financial and emotional support [2], which in many instances influences decisions related to healthcare seeking behavior, including during pregnancy [3]. However, men’s knowledge about maternal and child health is often limited due to the perception of reproductive and maternal health as a female domain [4, 5]. In the absence of sufficient knowledge about maternal health, often compounded by a lack of resources, male partners can negatively influence women’s engagement in perinatal health services, leading to detrimental impacts on health outcomes for the woman and child [6, 7]. Engaging men in maternal health services provides an important opportunity to increase knowledge and support for joint and informed decisions that positively affect the mother and child [2].

In sub Saharan Africa, maternal mortality has gradually reduced over time but still remains high, with a maternal mortality rate (MMR) of 510 deaths per 100,000 live births [8]. Similarly, despite declines in MMR in Tanzania, it remains at 410 deaths per 100,000 live births, nearly double the Millennium Development Goals (MDGs) target of 230 deaths per 100,000 live births [9]. After the MDGs were phased out, the Sustainable Development Goals (SDGs) were created with a new target of lowering the MMR to fewer than 70 deaths per 100,000 live births by 2030. Reaching this target will require increased efforts and support both nationally and globally [10]. A study in Tanzania showed that women who are married and/or living with a partner have an 85% increased risk of maternal death compared to those who do not; this counter-intuitive finding raises questions about the influence partners have on women’s care seeking behavior during pregnancy and childbirth [11].

In 2012, the Ministry of Health and Social Welfare in Tanzania released the National Plan for Eliminating Mother to Child Transmission of HIV (eMTCT) with the goal of eliminating new HIV infections among children [12]. Male involvement in antenatal care (ANC) is among the strategies recommended in this plan to help achieve the national eMTCT target. The guideline from this document (p.17) states: *“Male involvement will be prioritized through a multi-pronged approach in both the facility and the community. At the facility, couple HIV counseling and testing services will be offered and at the community there will be demand creation through village health committees and door to door HIV testing…”* [12]*.* Additionally, the national strategic plan for improving reproductive, maternal, newborn, child and adolescent health for 2016–2020 also recommends male involvement in reproductive health interventions, such as ANC, as a strategy to strengthen reproductive, maternal, newborn and child health [13].

The invitation of male partners to engage in ANC is a key entry point for them to partake in improving women’s reproductive and maternal health, including HIV outcomes [14]. Despite existing guidelines, male partner engagement in reproductive and maternal health in Tanzania is still fairly low. Data collected from prevention of mother-to-child transmission (PMTCT) of HIV programs in the country shows that only 30% of women present for couples counselling with their partners [13]. The literature has identified several barriers to men attending ANC with their pregnant partners, including a fear of HIV testing, poor knowledge about maternal health, and gender norms that prescribe men’s role as financial providers and perceive pregnancy as a female domain [15, 16].

Previous research has indicated numerous benefits to male partner engagement in ANC. Interventions that engage men in maternal healthcare have increased facility births and improved birth and complication preparedness and maternal nutrition [2]. The inclusion of men in ANC has also been shown to increase women’s acceptance of perinatal interventions [15], increase post-partum care visits and skilled birth attendance at delivery [17], and reduce the risk of mother to child transmission of HIV [18].

While the benefit of male engagement has been demonstrated, there is also emerging evidence that policies that enforce male engagement can have unintended consequences. Studies from East Africa suggest that the engagement of male partners in ANC may have a negative impact on women’s access to ANC services due to strategies used to implement the policy, such as denying care for women without partners and preferential treatment for women accompanied by partners [19–22]. Within the context of Tanzania’s national guidelines on male engagement, the objective of this paper is to explore how male partner engagement in ANC impacts women’s decision making to present to ANC, and their subsequent experience in ANC in urban health facilities in northern Tanzania.

## Methods

### Study design and setting

We conducted individual in-depth interviews with pregnant women and male partners who had attended a first ANC visit at one of two urban clinics in the Kilimanjaro region of Tanzania between June and July 2019. The two clinics provide routine HIV testing and counselling for pregnant women and their male partners. Both clinics follow the national guidelines for reproductive and child health and PMTCT. As such, they have both adopted approaches to increase the number of women who attend their first ANC visit with a male partner. Both clinics incentivize male attendance by prioritizing services for women accompanied by partners, questioning women attending without partners about partner absence, and encouraging women whose partners had not travelled to return home and convince their partner to attend with them. During the study period, about 53% of pregnant women in the two study clinics presented at first ANC with their male partner [23, 24].

### Sampling and recruitment

Study participants were selected from a sample of participants enrolled in a randomized control trial (RCT) of an HIV stigma reduction intervention [24]. Research assistants from the parent study recommended eligible participants based on the perception that they would speak willingly and freely about the research topic. Eligibility criteria included being 18 years of age or older, being a pregnant woman or an accompanying male partner presenting for a first ANC visit at one of the two study clinics, willingness to participate in an in-depth interview, and ability to provide consent. Participants were contacted by telephone and informed about the study; those who showed interest were scheduled for an in-depth interview. We purposively selected and contacted by telephone 10 couples and 10 women who presented for first ANC without a male partner. All 10 couples were willing to participate. However, three couples could not find time to come to the interview and one male partner failed to attend due to travel. Among women who presented without a partner, all agreed to participate but 4 women could not find time to attend the interview. Our final study sample consisted of 13 women and 6 male partners. Of the 13 female participants, 7 had presented for first ANC with a male partner and 6 had presented without a male partner. All 6 male participants had accompanied a pregnant spouse to a first ANC visit.

### Data collection

Individual in-depth interviews were conducted in a private office next to the study clinic and lasted between 30 min and 1 hour. Separate guides were used for male and female participants (see Additional files 1, 2 and 3). All interviews were conducted in Kiswahili by a Tanzanian male researcher (GAK), who has post-graduate training in qualitative methods. Interviews were audio recorded with consent of the participant. The interviewer used a semi-structured guide, which included opening questions and follow-up probes. Interview guides were developed based on the framework of the Social Relations Approach to gender and development planning [25, 26].

The Social Relations Approach provides a framework to examine the interactions among key institutions (e.g. household, community, state), and provided a useful structure to examine how governmental guidelines, clinic protocols, individual decision making, and intimate partner relationships interacted to influence women’s experiences in ANC with or without a male partner. The framework was utilized to probe how participants perceived each of these institutional levels influencing their decision-making process leading up to ANC, and the experience of ANC itself (See Table 1).

### Data processing and analysis

All audio recordings were transcribed and translated into English for analysis. A native Kiswahili speaker (HO) read through a few selected transcripts to check for accuracy. Any doubts regarding translation during analysis were discussed with two native speakers (HO and GAK). Data were analyzed using applied thematic analysis, which is a rigorous and systematic approach to identifying empirically driven themes in qualitative data [27]. Two researchers (HO and SSS) read the interview transcripts multiple times and wrote qualitative analytical memos for each individual interview [28]. Memos were structured based on the a priori domains of the interview guides. In each memo, inductive themes were summarized under each domain, and illustrative quotes highlighted key findings. Each memo was reviewed alongside the original transcript and discussed with another researcher (GAK or JNC) for concordance before being finalized. In examining coded text, the original transcript text was reviewed alongside memos to ensure consistency. The memos were approximately 6 pages (single spaced) each, representing a total of 114 pages of text across the 19 interviews. Memo-writing helped to identify emerging themes, which assisted in the development of a codebook. Afterwards, all memos were uploaded to NVivo12 [29] for coding and analysis. The applied thematic analysis [27] followed three steps. Firstly, memos were coded with broad themes that summarized the context of the pregnancy, women’s decision-making regarding presentation to ANC, their first ANC experience, and concerns regarding the level of male partner involvement in ANC. Secondly, major themes were further coded with inductive sub themes. Thirdly, reports of each code in NVivo (coded text and any annotations) were generated and exported from NVivo. Each report was studied for further synthesis of the findings and to draw deeper meaning from each theme.

### Ethical approval

We obtained ethical clearance from the National Health Research Committee of the National Institute for Medical Research in Tanzania (reference no. 2183) and the Kilimanjaro Christian Medical Centre (reference no. 915), as well as from our U.S. collaborating institutions (Duke University, D0371 and University of Utah, 00127605). All participants provided written informed consent before participating in in-depth interviews.

## Results

The average age of participants was 28 years (range 19–39) for pregnant women and 33 years (range 25–49) for male partners. Among pregnant women, nine were married, three were cohabiting, and one reported being unmarried. Eight out of the 13 pregnant women reported that their pregnancy was unplanned. Table 2 shows individual participant demographics.

**Table 1:** Content of the interview guide mapped onto the Social Relations Approach

Interview Guide Domain	Social Relations Approach – Key Institution	Interview Guide Questions
**Context of relationship and current pregnancy**	Family/kinship	History of relationship; marital and cohabitation status; planned or unintended pregnancy
	Family/kinship Community	Distribution of household responsibilities
	Family/kinship Community State	Role of partners in the acquisition and allocation of family income and other resources
**Decision-making and planning leading up to the first ANC visit**	Family/kinship	Events that led to the decision of attending ANC
	Family/kinship Community State	Barriers/facilitators for man and woman to attend ANC
**Experience during and directly after the first ANC visit**	Family/kinship	Fears, concerns, and expectations of attending the first ANC visit
	Family/kinship Community	Experience with HIV testing and counseling
	Family/kinship Community State	Perceptions of male partner’s role during the visit

Overall, we found that male partner engagement impacted women who were not accompanied by partners to the first ANC visit in two main realms: their presentation to ANC and their first ANC experience. For women who were accompanied by their partners to their first ANC visit, we found that they had concerns about the extent to which their partners were engaged in care.

### Presentation to care

We defined women’s presentation to care as their decision-making process of when to present for their first ANC visit. We identified two themes highlighting how male engagement guidelines are a challenge to women’s presentation to care: 1) women delayed presenting to care due to fears of the consequences of partner absence, and 2) women presented to ANC but were denied care due to the absence of their partner. As a result, the expectation of presenting to care with a male partner was an added stressor to women’s decision-making process to start ANC.

### Delays in presentation to care

Delays in presentation to care were common among women who knew they could not be accompanied by their partner or whose partners were indecisive about accompanying them to the clinic. Some women reported delaying to present to care because of waiting for their partner to be ready or willing to accompany them to the clinic. In this group, women reported waiting for their partner to complete other responsibilities in order to make time for ANC. One woman shared that although her husband was living in another town, she wanted to wait for him to come back so that they would attend together. However, he later decided that she should go without him in order to present early:

‘[My husband] is away for school. My [reason for] not coming [to the clinic] was not because I did not want to come at all, I wanted to wait until he was finished with school so that we could come together. But he told me a lot of time would have passed then, so it would be better if I came early [by myself].’ (Female, 23, partner did not present)

Some women delayed presenting to care because of waiting for a partner who seemed reluctant to accompany them to ANC. One man explained that he had tried to insist that his partner go to the clinic alone, with no reason as to why he could not accompany her. However, she insisted that she would not go without him. By the time he agreed to accompany her to the clinic, she was already 6 months pregnant.

‘About five or six months passed. Because each time I told her to go to the clinic, she would tell me that she wanted me to come with her. When I told her to go, she insisted that she wanted me to go with her…She was getting sick often and I would tell her that she should go to the clinic to check and see, maybe she had a UTI. But she told me that even if she went to the hospital, she wouldn’t get treatment [without a partner]. Therefore, she pestered me until the day I decided to come with her.’ (Male, 32, presented as partner)

### Denial of care due to partner absence

Women’s fears of being sent home due to partner absence were validated in the shared experiences of women who were denied care due to partner absence. Some women who presented to care without a male partner reported that clinic nurses refused to attend to them and asked them to go back home and return with their partner. One man described how his wife initially presented without him and was turned away; when he realized this, he decided to accompany her to the clinic.

‘When she came here [to the clinic], they told her that they were not going to test her [for HIV] without her husband. Therefore, that is where the conversation began. I asked her about the day of the appointment for going back to the clinic, and she told me the day, and I agreed to come. When the time arrived, I came with her.’ (Male, age unknown, presented as partner)

For women who were unable to present with a partner, the requirement of male accompaniment was an added stressor to their decision of when to present to care. Women who were unable to present with a partner were asked to bring a letter from their village/street chairperson to confirm the absence of their partner. This further delayed women’s presentation, and some women even chose not to return to the clinic. One woman who was unable to present with a partner recalled her experience:

‘I came the first time and they sent me back, telling me that I should go and bring my partner, and I couldn’t explain myself. When I left, I came a second time and I told them that my partner was far away, and she (the nurse) told me that if my partner was not around, I should go and bring a letter from the chairman, and I went back home. When I went back home, I didn’t even go to the chairman, I decided to stay at home. But later on, I thought that the time was advancing, and I went back to the clinic but that day they just gave me the services without asking me about my spouse.’ (Female, 23, partner did not present)

### First ANC experience

The expectations of male attendance impacted women’s experiences at their first ANC visit in three main ways: being questioned about an absent partner, differential treatment due to partner absence, and dissatisfaction with how male partners were involved in care.

### Inquiries about partner absence

Women who presented without a partner described being questioned by nurses about their partner’s whereabouts. Although some women reported that nurses understood and accepted their situation, others described these inquiries as stressful, and noted that it was a source of distress and anger.

‘[The nurses] asked me where my husband was and I told them that my husband was far away, that he worked far away. They asked me how it was that I came on my own. [They said] that they wanted to know what my HIV status was, and that they couldn’t test me alone. The nurse did not understand me, and I was angry and I went outside to call my husband on the phone. I told him that they had refused to give me services and I didn’t know what to do about it. …. I also talked to my mother and she advised me that I shouldn’t try to find someone else to stand in for my husband, that I should talk to my husband so that he could come to test so that we could know about our health. I talked to him again, tried to make him understand and later I went back to talk to the nurse. And after I had made her understand [that my husband couldn’t attend] she agreed to test me.’ (Female, 32, partner did not present)

Our data suggests that the onus of male presentation at the clinic fell solely on the women. Women in the study described how they were questioned about their partners’ absence. None of the pregnant women mentioned the healthcare staff contacting their partners to inquire about their absence or encourage them to present to the clinic for first ANC.

### Being treated differently due to partner absence

Women who received services despite the absence of their partner reported being treated differently than those presenting with a partner. Clinic nurses were reported to provide preferential treatment to women who presented with a partner by attending to them first. Those without partners were reported to be normally attended to last, even if they arrived at the clinic earlier. Preferential treatment was used to incentivize women to present to the clinic with their partner, causing women who presented without partners to spend more time waiting at the clinic.

‘The difference is that those who had come with their spouses were given priority in receiving the services so that their men would be able to go back to their responsibilities early … Those of us who had come without our husbands had to wait until the others had finished.’ (Female, 33, partner did not present)

Another woman who presented without a partner echoed a similar sentiment of receiving differential treatment:

'It is different because first, you are discriminated. You may be put last even if you arrived first.' (Female, 32, partner did not present)

### More focus on HIV testing and less on ANC

Although women who presented with partners reported being generally satisfied with the service at the clinic and experienced very little trouble with how male engagement was implemented, some shared that they had expected their partners to participate in pregnancy-related activities and not just in HIV testing. One woman who shared this sentiment explained:

‘I think that he should have been involved in everything so that he would know these things...I felt as if they just wanted him to come and test and then leave.’ (Female, 19, partner presented)

## Discussion

Our findings show that policies and practices that mandate male engagement in ANC impact women’s initial presentation to ANC and influence the care they receive during their visit. Despite the well-intentioned goals of male engagement guidelines, we observed that these clinic practices led to women having delayed presentation to care, which in turn can impact health outcomes. The implementation of these guidelines also disadvantages women presenting without partners due to their differential treatment, including possible denial of care and delays in receiving care as a result of the prioritization of women who present with partners. Although male engagement in ANC is meant to improve care for all women and their partners, strategies used to implement it make ANC less accessible to women unable to present with a partner. These unintended consequences could outweigh the benefits that male engagement in ANC is meant to offer. This study adds to evidence that the implementation of male engagement in ANC in Tanzania could create inequities in care and disadvantage women who are not in stable relationships [19, 20, 22, 30].

According to the focused ANC guidelines, it is recommended that women present for their first ANC visit during the first 12 weeks of their pregnancy [31]. Our findings, together with another study in southern Tanzania [20], show that women whose partners are reluctant to accompany them to the clinic may delay presenting for care, sometimes until the last trimester of pregnancy. This is important to consider in the context of unplanned pregnancies, as women with unplanned pregnancies are already more likely to have a delayed presentation to ANC for numerous reasons [32]. Late presentation to ANC poses great risk among expectant mothers as they miss out on important care provided at ANC including vitamin supplements, regular vaccination and regular monitoring of the growth of the fetus [33]. In Tanzania, only 15% of women attended ANC during the first 16 weeks of their pregnancy [13], due to barriers such as long distance from the clinic and cultural beliefs that discourage revealing an early pregnancy. Fears of being turned away from the ANC due to partner absence, coupled with pre-existing barriers to early attendance of ANC, are likely to cause more women to delay clinic attendance, and therefore play a role in increasing the likelihood of poor maternal and child health outcomes.

In sub-Saharan Africa, men have lower rates of uptake of HIV testing compared to women [34]. Consequently, male involvement in ANC is a key strategy used to increase routine HIV testing among men in the region [35, 36]. Studies from sub-Saharan Africa show that fear of an HIV-positive diagnosis is a barrier for uptake of testing among men [37, 38], and this fear also contributes to men’s hesitation to attend a first ANC visit where HIV testing is a requirement. When women are required to bring their male partner to their first ANC visit, it puts an unfair burden on pregnant women. Our findings do not point to any efforts made by healthcare workers to directly contact male partners in an effort to invite or motivate them to attend first ANC with their partner, or to investigate their reluctance to do so. Strategies to increase male HIV testing within and outside of the ANC setting, such as providing letters of invitation for men to attend ANC [39], encouraging and providing access to self-testing [40], provision of mobile and home based testing [41], and creating more ‘male friendly’ environments in health centers for example, allocating waiting areas for male partners [42, 43], could help reduce the cost that women incur for the absence of a male partner at first ANC.

Our study supports others in raising questions about whether male engagement is being used to its full advantage of bringing in men as partners in maternal health [19]. Several women in our study expressed dissatisfaction with the extent to which their partners were engaged in their ANC. They noted that their partners were required to present primarily to participate in HIV testing and counselling and were not fully engaged in other aspects of the visit that were pertinent to pregnancy care. The over-riding focus for men attending is dominated by the need to test them for HIV; this presents a missed opportunity to engage men more broadly in ANC and post-partum care of both the mother and the child. A study in Rwanda reported similar findings; although male partners were required to present for the first ANC visit, male engagement was primarily considered to be for HIV testing, and any further male involvement after the first ANC visit was discouraged by health workers [21]. It is important to ensure that male engagement in ANC is implemented to benefit the woman and her partner by involving him in educational components of care and utilizing the visit to encourage men to support their partners in accessing other reproductive healthcare services during pregnancy, labor and delivery, and the postpartum period [17, 44].

This study found that preferential treatment was given to women who presented with a male partner, even though this was not a practice that was recommended in the national plan. Preferential treatment has also been documented in Southern Tanzania [22] and in Rwanda [21] as one of the strategies health care workers use to encourage men to attend ANC, especially when clinics are required to report on male attendance during ANC and the number of male partners tested for HIV. While this strategy was presented as a pragmatic action to allow men to return to work, it is imbedded in a false narrative that men are the sole contributors to household income, and it discriminates against women attending without partners. This is evident in our findings, as our study sample shows that nine out of the thirteen female participants reported that they contributed to household income (see Table 2). Such strategies to encourage male partner accompaniment may deter women without supportive partners from attending ANC due to fear of stigma and frustration with delayed services. In addition, denial of care to women presenting without a partner and other discriminatory strategies used by health workers could potentially undermine women’s trust in the healthcare system and in turn, negatively impact women’s future care engagement in ANC [30], PMTCT [45] and delivering in health facilities [46]. While the implementation of male engagement guidelines is important, health workers should be mindful of how strategies used during implementation may affect the quality and experience of care among women presenting without a partner and how this could ultimately influence them to delay seeking care or opt to disengage in care altogether.

**Table 2:** Participant demographics

ID	Age (years)	Sex	Relationship status	Occupation	Education	Planned pregnancy	Attended with Partner
01	32	Female	Cohabiting	Employed	College	Yes	No
02	23	Female	Unmarried	Housewife	College	Yes	No
03	25	Female	Married	Self-employed	College	No	No
04	26	Male	Cohabiting	Self-employed	College	N/A	N/A
05	29	Female	Cohabiting	Self-employed	Secondary	No	Yes
06	33	Male	Unmarried	Self-employed	Primary	N/A	N/A
07	19	Female	Married	Self-employed	Secondary	No	Yes
08	25	Male	Unmarried	Self-employed	Secondary	N/A	N/A
09	37	Female	Married	Housewife	Primary	No	Yes
10	49	Male	Married	Employed	Primary	N/A	N/A
11	32	Female	Married	Self-employed	Primary	Yes	No
12	33	Female	Married	Self-employed	Primary	No	No
13	28	Female	Married	Self-employed	College	Yes	No
14	23	Female	Cohabiting	Housewife	Primary	No	Yes
15	25	Male	Cohabiting	Employed	Secondary	N/A	N/A
16	39	Female	Married	Self-employed	Primary	No	Yes
17	39	Male	Married	Peasant Farmer	Primary	N/A	N/A
18	19	Female	Married	Housewife	Secondary	No	Yes
19	19	Female	Married	Peasant Farmer	Primary	Yes	Yes

In light of our findings, there is value in educating healthcare workers and the wider community about the unintended consequences of requiring male partner attendance, and how they could be addressed. Health workers and hospital leadership should be made aware of the challenges that women face while seeking care and how the strategies used affect women’s access to care and may in turn affect pregnancy and birth outcomes. These conversations could be facilitated through sharing findings from studies that report on this topic, or more directly by encouraging dialogue between women and care providers [47, 48]. Clinic staff and hospital leadership could also benefit from training on evidence based strategies, specifically those mentioned earlier [39, 42, 43],which could be used to promote a more holistic approach for male engagement in ANC [49]. Sensitization among male partners and the wider community on the benefits of male engagement in ANC and postpartum care overall is crucial for successful implementation of these guidelines. This could be achieved through community sensitization campaigns with targeted content to motivate men [50], engaging in community dialogue [47, 48], and the use of mass and social media [51]. The existing body of evidence on the effects of the implementation of male engagement guidelines supports the need for a revision of these guidelines so that they do not disadvantage women. Revision of the guidelines would benefit from evidence based strategies to engage men in ANC and to broaden guidelines to reflect a more holistic engagement of men in ANC and postpartum care [49], for example, by encouraging male engagement beyond HIV testing and supporting male attendance in subsequent ANC visits.

### Limitations

The results of this study must be considered in light of its limitations. This study included two urban clinics that see a high volume of pregnant women; however, these clinics might not be representative of other clinics in the region. Given that we recruited participants from a larger randomized control trial, purposively selecting participants who could contribute rich information, we may have missed participants who had a different set of perspectives and experiences with the research topic. There is also potential for a social desirability bias among participants, especially since they were asked to report on services they received at the clinic. To address this, we assured participants that the information they shared would not affect the care or services they receive at the clinic. We did not conduct interviews with clinic nurses regarding the strategies they use in the implementation of this policy in the clinic setting, which would triangulate data from patients. However, in a recent study in southern Tanzania, healthcare workers confirmed the use of strategies such as denying women care in order to influence them to bring their partners to the clinic [22]. Future studies should include interviews with health care workers in ANC clinics, which could assist in putting women’s reported experiences into perspective.

## Conclusions

The findings reported in this study explore the impact of male engagement guidelines on pregnant women’s ANC attendance and their experiences in care in an urban setting in Tanzania. While male engagement in ANC can benefit maternal and child health, results demonstrated that the implementation of guidelines intended to promote male partner participation may have unintended consequences for women, including delayed presentation to ANC, delays in receiving care at the clinic, and disrespectful care. Future strategies may include incentives for male engagement such as letters of invitation [39] and creating a more male friendly environment in the ANC setting [42, 43]. Although HIV testing and counselling is very important, the implementation of this policy should encourage the full engagement of male partners in antenatal care and education for the benefit of the woman, her partner and their unborn child.

## Supplementary Information

**Additional file 1.** In-depth interview guide for male partners.

**Additional file 2.** In-depth interview guide for women presenting with partners.

**Additional file 3.** In-depth interview guide for women presenting without partners.

## Data Availability

The qualitative datasets generated and analyzed in this study are not publicly available because study participants did not provide consent for their information to be shared publicly. Summaries of the data are available upon request to the corresponding author.

## Abbreviations

ANC: Antenatal Care
RCT: Randomized control trial
MDG: Millennium Development Goals
SDG: Sustainable Development Goals
EMTCT: Elimination of Mother-to-Child Transmission of HIV
PMTCT: prevention of mother-to-child transmission of HIV
