# Supplementary material for: Male engagement guidelines in antenatal care: unintended consequences for pregnant women in Tanzania
Source: BMC Pregnancy Childbirth. 2021 Oct 26;21:720. doi: 10.1186/s12884-021-04141-5 (PMC8549379; doi:10.1186/s12884-021-04141-5)
Supplement: Supplementary file 1 — Additional file 1. In-depth interview guide for male partners. [file 12884_2021_4141_MOESM1_ESM.pdf]

Male engagement guidelines in antenatal care: Unintended consequences for pregnant women in Tanzania

Authors:

Haika Osaki

Saumya S. Sao

Godfrey A. Kisigo

Jessica N. Coleman

Rimel N. Mwamba

Jenny Renju

Blandina T. Mmbaga

Melissa H. Watt

IN-DEPTH INTERVIEW GUIDE FOR MALE PARTNERS

Thank you for agreeing to spend some time talking with me today. Our conversation will take about an hour, and the tape recorder will be used to make sure that nothing will be missed during our interview. What you say to me today will be confidential, and I will not share any information with your health care providers. The purpose of these interviews is to understand the experiences of men in antenatal care, so that we can support them better. I hope you will feel comfortable to speak freely and honestly with me. Do you have any questions or concerns before we begin?

I. Introduction

I know that you've had a chance to talk to other members of our research team during our study. I'd like to start by getting to know you better. Can you tell me a little about yourself?

> [NOTE: Give him the opportunity to talk briefly about himself. Let him know that you'll come back to some of these issues later in the interview.]

Can you tell me more about your relationship with your partner?

- History of relationship, living together or not, other children
- Probe here to ask how resources and responsibilities are divided between the partners:
  - Does he have a source of income?
  - Does his partner have a source of income?
  - How is household work shared between the two of them?
  - Who controls the income of the family? Does he give his partner money (for example, for kids, for antenatal care)?

II. Index Pregnancy

I'd like you to think back to when you first found out your partner was pregnant.

How did you learn she was pregnant?

- Was it planned?
- How did she tell you she was pregnant? What was your response?
- Do you think it was hard for her to tell you? Why/why not?

How are you feeling about the pregnancy?

- General feelings about his role in the pregnancy, fears or concerns about having a child
- Any previous experiences with pregnancy/children

III. Decision-making and planning for 1st ANC appointment

Now think about when you and your partner were first deciding to attend ANC.

Can you tell me a little bit more about the decision to attend your first ANC visit?

- Were you involved in making the decision?
  - Did your partner discuss attending ANC with you? Did you bring up attending ANC at all?
    - If so, how did that conversation go?
    - Were you comfortable discussing ANC with your partner? Why?

- What caused your partner to come to ANC?
- What were the main reasons you decided to attend ANC?

Tell me why you decided to attend this clinic (Majengo/Pasua) for ANC care.

- Did you have to overcome any barriers to get to this clinic for ANC care?
    - Probes: taking time off work, fear of feeling uncomfortable in clinic, costs of transportation, any relationship factors?

If barriers: Who helped you to overcome these barriers?

IV. 1st ANC Appointment Experience

Think back to the day of the appointment. Can you tell me how you were feeling?

- Probes:
    - Fears**,** concerns, expectations

Now I would like to hear about what happened during that first visit. (*Have him walk through the visit before asking any probing questions about the HIV test*)

Of course, HIV testing is a big part of the first ANC visit. I'd like to hear more about the experience you and your partner had getting an HIV test.

- Did you receive counselling prior to the HIV test?
    - Was this counselling just with your partner, or also with other people?
    - What did the nurse talk to you about before doing the test?
- Did you and your partner test together? **Did you receive the results of your HIV test together?**
    - What did the nurse talk to you about after you received your results?
    - How did you feel receiving the test results with your partner/alone? (whatever happened)
    - How would you have felt if you received the results alone/together (the **opposite** of what he says happened)?
    - How do you feel that taking an HIV test and receiving the results of an HIV test with your partner is good or bad?

What do you think your role was during that first ANC visit?

- Did you ask questions, did the nurse ever speak to you directly?
- Were you satisfied with your role during that visit, or do you want it to be different?
- How do you think your partner felt about you being at the visit?

Beyond 1st ANC appointment, how do you think men should be involved in their partner's ANC, delivery, and postpartum care with your partner?

- After that first ANC appointment, did you discuss ANC care, or pregnancy-related concerns, with your partner? (What did you discuss?) Did you discuss with any other family members?
- What kinds of support does your partner expect during this pregnancy?
    - What kinds of support are you willing to provide your partner during pregnancy?

V. Perceptions of Male Engagement in ANC

Why do you think clinics/government want men to come to ANC?

- Do you agree about the requirement for men to attend first ANC appointment? Why / why not?

- What do you think the impact of men coming to ANC would be for the woman?
- How do you think your attendance improved or worsened your partner's ANC?

Do you think men *want* to come to the ANC visit with their partners?
- Why / why not?
- Which types of men might be more or less willing to join their partners at ANC?
    - May it depend on HIV risk, partner stability?

What obstacles do you think men face in general in attending ANC?
- Logistical issues (e.g., time off work)
- Social norms (gender norms, embarrassment, clinic as a "women's space")
  What about the ANC environment makes it comfortable/not comfortable for men?

Now, take some time to summarize the interview to this point.
Example: What I'm hearing from you is that you think men are asked to come to ANC in order to test for HIV, and that makes men afraid to attend. Is that right?

Probes:
- Other than the HIV test, do you think there are <u>other</u> reasons why men should attend ANC with their partners?
- Do you think the focus on HIV test might make it harder for men to be engaged in ANC more generally?
- What could be done about that?

VI. Opportunities to improve male engagement in ANC

What do you think might help men to come to ANC with their partners and be involved in the pregnancy?

What could be done to make men feel more comfortable with HIV testing?
- Education or other campaigns
- Testing in other places (work, community)

Thank you for everything you have shared with me. Is there anything else you would like to add?
