# Supplementary material for: Male engagement guidelines in antenatal care: unintended consequences for pregnant women in Tanzania
Source: BMC Pregnancy Childbirth. 2021 Oct 26;21:720. doi: 10.1186/s12884-021-04141-5 (PMC8549379; doi:10.1186/s12884-021-04141-5)
Supplement: Supplementary file 3 — Additional file 3. In-depth interview guide for women presenting without partners. [file 12884_2021_4141_MOESM3_ESM.pdf]

Male engagement guidelines in antenatal care: Unintended consequences for pregnant women in Tanzania

Authors:

Haika Osaki

Saumya S. Sao

Godfrey A. Kisigo

Jessica N. Coleman

Rimel N. Mwamba

Jenny Renju

Blandina T. Mmbaga

Melissa H. Watt

IN-DEPTH INTERVIEW GUIDE FOR WOMEN PRESENTING WITHOUT PARTNERS

Thank you for agreeing to spend some time talking with me today. Our conversation will take about an hour, and the tape recorder will be used to make sure that nothing will be missed during our interview. What you say to me today will be confidential, and I will not share any information with your health care providers. The purpose of these interviews is to understand the experiences of women and men in antenatal care, so that we can support them better. I hope you will feel comfortable to speak freely and honestly with me. Do you have any questions or concerns before we begin?

I. Introduction

I know that you've had a chance to talk to other members of our research team during our study. I'd like to start by getting to know you better. Can you tell me a little about yourself?

> [NOTE: Give her the opportunity to talk briefly about herself. Let her know that you'll come back to some of these issues later in the interview.]

Can you tell me more about your relationship with the father of your child?

- History of relationship, living together or not, other children
- Probe here to ask how resources and responsibilities are divided between the partners, or about her responsibilities if she does not have a partner:
  - household vs outside work
  - source of income; how much control she has over income
  - Who makes decisions in the relationship?
  - How well you communicate

After this point, refer to father of child as father of child or partner.

II. Index Pregnancy

I'd like you to think back to when you first realized you were pregnant.

How did you learn you were pregnant?

- Was it planned?
- Did you tell your partner you were pregnant? How did you tell your partner you were pregnant? (what was hard about this)
- What was his response?

How are you feeling about your pregnancy?

- General feelings about pregnancy, giving birth, having a baby
- Any previous experiences with pregnancy

III. Decision-making and planning for 1st ANC appointment

When did you come to the clinic for your first antenatal care visit? (how far into pregnancy)

Can you tell me a little bit more about your decision to attend your first ANC visit?

- What were the main reasons you decided to attend ANC?
- Who helped you decide to come to ANC? (male partner, family members, friends?)
  - Did you discuss attending ANC with your partner?
    - If so, how did that conversation go?

        - If no, would you feel comfortable discussing ANC with your partner? Why or why not?
        - ****Make sure here to probe because she did not bring her partner****
- **Why did your partner not come to ANC? **probe here – very important question****

Tell me why you decided to attend this clinic (Majengo/Pasua) for your ANC care.

- Did you have to overcome any barriers to get to this clinic for ANC care?
    - Probes: access to transportation, costs, childcare, partner-related barriers (perhaps she would have to ask partner for money for transport/care)

If barriers: Who helped you to overcome these barriers?

IV. 1st ANC Appointment Experience

Think back to the day of your first ANC visit. Can you tell me how you were feeling?

- Probes:
    - Fears**,** concerns, expectations

Now I would like to hear about what happened during that first visit. (*Have her walk through the visit before asking any probing questions about the HIV test*)

Of course, HIV testing is a big part of the first ANC visit. I'd like to hear more about the experience you and your partner had getting an HIV test.

- Did you receive counselling prior to the HIV test?
    - What did the nurse talk to you about before doing the test?
- Did you receive the results of the HIV test by yourself?
    - What did the nurse talk to you about after you received your results?
    - How would you have felt if you received the results with your male partner?
    - How do you feel that women receiving an HIV test with their partner is good or bad?

Did you feel that you were being treated differently or receiving different information than women who came with partners?

- Did you feel underserved because you did not have a partner?
- Did you feel any stigma associated with the fact that you showed up by yourself? Or that you are pregnant but don't have a partner to present with/are not married to the father of your child? ****probe here because this is important****
- Did you want your partner at that ANC appointment? Why?

Beyond the 1st ANC appointment, how do you think men should be involved in their partners' ANC, delivery, and postpartum care?

- After your first ANC appointment, did you discuss ANC care, or pregnancy-related concerns, with your partner? (What did you discuss?) Did you discuss with any other family members?
- How do you want your partner to support you during pregnancy, during your birth, and when the baby comes?
    - What have you two discussed about this?
- **If no partner:** where else can you find support during your pregnancy? (family members, friends?)

V. Perceptions of Male Engagement in ANC

Why do you think clinics/government want men to come to ANC?

- Do you agree about the requirement for men to attend first ANC appointment? Why / why not?
- What do you think the impact of men coming to ANC would be for the woman?
- If your partner had attended ANC, how do you think your ANC experience would have been better or worse?

Do you think men *want* to come to the ANC visit with their partners?

- Why / why not?
- Which types of men might be more or less willing to join their partners at ANC?
    - May it depend on HIV risk, partner stability?

What obstacles do you think men face in general in attending ANC?

- Logistical issues (e.g., time off work)
- Social norms (gender norms, embarrassment, clinic as a "women's space")
  What about the ANC environment makes it comfortable/not comfortable for men?

Now, take some time to summarize the interview to this point.
Example: What I'm hearing from you is that you think men are asked to come to ANC in order to test for HIV, and that makes men afraid to attend. Is that right?

Probes:

- Other than the HIV test, do you think there are <u>other</u> reasons why men should attend ANC with their partners?
- Do you think the focus on HIV test might make it harder for men to be engaged in ANC more generally?
- What could be done about that?

VI. Opportunities to improve male engagement in ANC

What do you think might help men to come to ANC with their partners and be involved in the pregnancy?

How do you think your ANC experience would have been different if your partner had come?

What could be done to make men feel more comfortable with HIV testing?

- Education or other campaigns
- Testing in other places (work, community)

Thank you for everything you have shared with me. Is there anything else you would like to add?
